# Cannabinoids Accumulation in Hemp (*Cannabis sativa* L.) Plants under LED Light Spectra and Their Discrete Role as a Stress Marker

**DOI:** 10.3390/biology10080710

**Published:** 2021-07-24

**Authors:** Md. Jahirul Islam, Byeong Ryeol Ryu, Md. Obyedul Kalam Azad, Md. Hafizur Rahman, Eun Ju Cheong, Jung-Dae Lim, Young-Seok Lim

**Affiliations:** 1 Department of Bio-Health Convergence, College of Biomedical Science, Kangwon National University, Chuncheon 24341, Korea; jahirulislam213@gmail.com (M.J.I.); fbqudfuf0419@naver.com (B.R.R.); azadokalam@gmail.com (M.O.K.A.); hafizknu94@gmail.com (M.H.R.); 2 Physiology and Sugar Chemistry Division, Bangladesh Sugarcrop Research Institute, Ishurdi, Pabna 6620, Bangladesh; 3 Division of Forest Science, College of Forest and Environmental Sciences, Kangwon National University, Chuncheon 24341, Korea; ejcheong@kangwon.ac.kr; 4 Department of Herbal Medicine Resource, Kangwon National University, Samcheok 25949, Korea

**Keywords:** hemp, light spectra, light stress, photosynthetic activities, antioxidant enzymes, antioxidant, cannabinoids and stress markers

## Abstract

**Simple Summary:**

Cannabinoids accumulation in the hemp plant greatly depends on light quality under a controlled growing system. Sativa-type hemp plant (enriched with THC) increased CBD accumulation under some controlled light combinations. Green light has a significant role in CBD and CBDA synthesis, where FR and UV-A (along with green) play a positive and negative role in this process, respectively. Earlier, cannabinoids were identified as stress markers, but it was unclear which compound/compounds are directly involved with the light stress environment as stress markers in the hemp plant. In our study, THCA showed a significant role as a stress marker followed by CBDA. On the other hand, THC and CBD showed a negligible response as stress response compounds to such conditions.

**Abstract:**

Hemp adaptability through physiological and biochemical changes was studied under 10 LED light spectra and natural light in a controlled aeroponic system. Light treatments were imposed on 25 days aged seedlings for 16 h daily (300 µmol m^−2^ s^−1^) for 20 days. Plant accumulated highest Cannabidiol (CBD) in R_7_:B_2_:G_1_ light treatment, with relatively higher photosynthetic rate and lower reactive oxygen species, total phenol content, total flavonoid content, DPPH radical scavenging capacity, and antioxidant enzymatic activities. Tetrahydrocannabinol (THC) also accumulated at a higher level in white, R_8_:B_2_, and R_7_:B_2_:G_1_ light with less evidence of stress-modulated substances. These results indicated that CBD and THC have no or little relation with light-mediated abiotic stress in hemp plants. On the contrary, Tetrahydrocannabinolic acid (THCA) was accumulated higher in R_6_:B_2_:G_1_:FR_1_ and R_5_:B_2_:W_2_:FR_1_ light treatment along with lower photosynthetic rate and higher reactive oxygen species, total phenol content, total flavonoid content, DPPH radical scavenging capacity, and antioxidant enzymatic activities. However, Cannabidiolic acid (CBDA) was accumulated higher in R_6_:B_2_:G_1_:FR_1_ light treatment with higher stress-modulated substances and lower physiological traits. CBDA was also accumulated higher in R_8_:B_2_ and R_7_:B_2_:G_1_ light treatments with less evidence of stress-modulated substances. Besides, Greenlight influenced CBD and CBDA synthesis where FR and UV-A (along with green) play a positive and negative role in this process. Overall, the results indicated that the treatment R_7_:B_2_:G_1_ enhanced the medicinal cannabinoids most, and the role of THCA as a stress marker is more decisive in the hemp plant than in other cannabinoids under attributed light-mediated stress.

## 1. Introduction

Light is the primary source of energy for plant growth and development through photosynthesis. This growth and developmental process depend on light spectral quality, intensity, compositions, duration, and direction [1]. A small irradiance of light can bring changes in several compositions in growing plants [2]. These processes come through light interaction with species and cultivars, which mainly depend on its irradiation, enhancing stressful or non-stressful events for plants [3]. Despite energy sources for photosynthesis, light can simultaneously act as a stress factor as plant response to light mainly depends on the lighting environment, genotypes, cultivation practices, etc. [4]. Excess light (irrespective of temperature effect) increases evaporation and photoinhibition, resulting in dehydration in leaf tissue, causing reduced photosynthetic production [5].

Plants respond differently to each spectral band of light. Plants use the wavelength of red light to accumulate carbohydrates and nutrients [6,7], red and blue for electron excitation in the photosynthesis process [1,8] and, blue and UV to synthesize carotenoids and anthocyanins [9,10]. Evidence showed that high intensities of UV-B radiation cause stress to plant by inducing DNA damage, photoinhibition, lipid peroxidation, and finally, growth retardation [11]. When plants are exposed to such light intensities or any abiotic stress condition, the demand for metabolic processes in carbon fixation increases for energy supply and reduces power by involving photosystems and electron transport chains [12,13]. This asymmetry generates reactive oxygen species (ROS), which have both signaling and toxic (oxidative damage by inducing lipid peroxidation) effects on cells [1,14]. Light intensity below the compensation point also will result in a net loss of photosynthetic products, and more light after saturation point has no or negative effect on photosynthesis [7,15].

Bioactive compounds are collectively known as primary and secondary metabolites, which gave aroma, color, taste even provide resistance against external biotic and abiotic stress [16]. Evidence proved that different forms of external stress help plants produce bioactive compounds [17,18,19,20,21,22]. Previous studies suggested that the R:FR ratio, the blue and UV light photoreceptors (CRYs, PHOTs, and UVR8), can alter signaling pathways. These changes may affect phytohormone-mediated regulation of growth, development, physio-biochemical pathways, finally, plant root architecture [23,24,25], which may create partial water stress to plant.

It was reported that despite the negative effect on quantum yield, plants attained a higher photosynthetic rate and biomass accumulation under supplemental UV-A radiation. This higher photosynthetic rate was due to an increase of stomatal conductance (gs) instead of the ratio of intracellular to ambient CO_2_ content (Ci/Ca) [26]. Besides these, ultraviolet light has a crucial role in plant response to several morphological, physiological, and secondary metabolites production, which are combinedly termed as plant photomorphogenic response [27,28,29,30]. These photomorphogenic responses mainly controlled by UVR8 by regulating gene expression relate to hypocotyl elongation inhibition, DNA repair, antioxidative defense, and phenolic compounds production [31]. On the other hand, far-red (710–850 nm) may have an essential role in photosynthetic purposes in leaves [32]. A high or low R and FR light ratio change the mode of action in phytochromes, converting the Pr into Pfr, or vice versa [20]. This conversion may bring changes in gene expression related to photomorphogenesis [33,34,35]. However, the role of UV-A and FR with the combination of other spectral bands relate to plant physiology, and morphological changes remain poorly understood.

Hemp is an annual herb belongs to the Cannabaceae family has been exploited for medicinal purposes for more than 10,000 years [36]. Living hemp plants contain cannabinoids as carboxylic acid like THCA and CBDA that decarboxylate during storage and heating transform to neutral cannabinoids such as THC and CBD [37,38,39,40]. Although secondary metabolites in cannabis are mostly controlled by selecting genotypes and their phenotypic characteristics; however, some horticultural techniques, including photoperiod, lighting intensity, and quality, can change among them [37,41,42,43]. Earlier in a study, the increment of THC in cannabis was described when it was treated under controlled UV-B radiation [41]. Ning et al. showed that UV-A and UV-B could increase secondary metabolites in Lonicera japonica medicinal plants [44]. For this reason, the plant treated with low dose UV mediated stress is crucial from a biotechnological and pharmaceutical point of view to increase valuable compounds [45]. Previous studies revealed that under long-time UV treatment, all types of cannabinoids did not respond equally [41,46]. It is also essential to find out the LED combination that can manipulate different targeted cannabinoids compounds by bringing in a metabolic system change in hemp plants. Besides, it is not clear which cannabinoids are directly involved with a light stress environment identified as stress markers in the hemp plant. Therefore, the objectives of the study were to determine the suitable LED combination for the higher accumulation of medicinal cannabinoids and select the stress markers of cannabis plants under light-mediated stress conditions.

## 2. Materials and Methods

### 2.1. Experimental Design and Treatment

**Figure 1:**
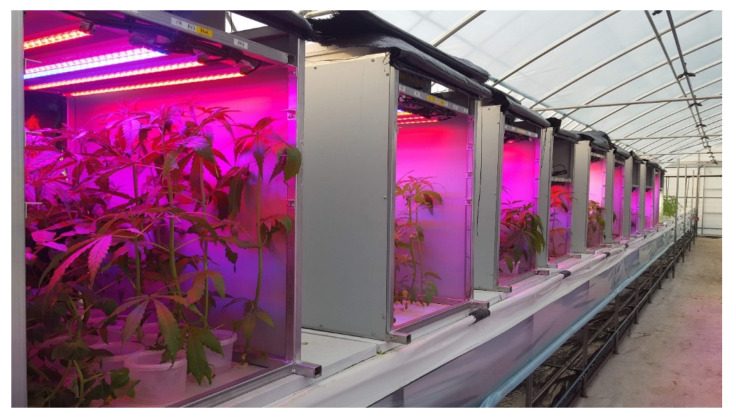
Hemp plant under treatment in steel made chamber (80 cm × 60 cm × 80 cm) equipped with different LED light. Here and subsequent models: L1, Natural light; L2, White; L3, R_8_:B_2_; L4, R_7_:B_2_:G_1_; L5, R_7_:B_2_:FR_1_; L6, R_6_:B_2_:G_1_:FR_1_; L7, R_5_:B_2_:W_2_:FR_1_; L8, R_5_:B_2_:G_1_:FR_1_:UV_1_; L9, R_6_:B_2_:FR_1_:UV_1_; L10, R_4_:B_2_:W_2_:FR_1_:UV_1_; L11, R_2_:B_2_:G_2_:W_2_:FR_1_:UV_1_. All treatments used a photosynthetic photon flux density of 300 µmol m^−2^ s^−1^.

Hemp seeds (*Cannabis sativa* L. strain India) were sown in sixteen cells plug tray (27 cm× 27 cm × 6 cm) filled with commercial soil mixture (Bio-soil No. 1, Heungnong Agricultural Materials Mart, Korea) in a glasshouse. Before sowing, the seeds were sterilized [70% (*v*/*v*) ethanol, 0.1% (*w*/*v*) HgCl_2_ and 0.2% (*w*/*v*) thiram] and soaked in water for 24 h at room temperature to facilitate the germination. The environmental conditions such as temperature, relative humidity (RH), and photoperiod were recorded at 30/25 °C (day/night), 60–70%, and 12 h, respectively. The seedlings were irrigated daily using tap water to the field capacity level. After three weeks, the seedlings were transferred/transplanted to the steel made chamber (80 cm × 60 cm × 80 cm) to adjust with the nutrient solution. After one week of adjustment, the plants were subjected to treatment with different LED light (Bisol LED light Co., Seoul, Korea) combination (Figure 1; Table 1). The photosynthetic photon flux density (PPFD), photoperiod, and temperature of the chamber were 300 µmol m^−2^ s^−1^, 16 h (6.00 AM to 10.00 PM), and 23 to 27 °C, respectively. The PPFD was checked and adjusted at the top leaf-level every other day. The plant chamber was designed for an aeroponic system where nutrient formulated water (Table 2) was sprayed to the plant root zone for twenty seconds every two minutes. After 20 days of LED treatment, the youngest completely formed leaves were collected as samples from several randomly selected plants as biological replications subjected to further analysis.

### 2.2. Leaf Gas Exchange Measurement

The net photosynthetic rate (A, µmol m^−2^ s^−1^), transpiration rate (E, mmol m^−2^ s^−1^), stomatal conductance (gs, mmol m^−2^ s^−1^) were measured on well-developed leaves (3rd node from the top) of six plants under each treatment using an LCpro gas analyzer (ADC BioScientific Ltd., Hoddesdon, Herts EN11 ONT, UK). The level of A, gs, E, and WUE was measured at the ambient environmental condition. The measurements of gas exchange were carried out at the mid-day between 10.00 AM and 3.00 PM. The photosynthetic water use efficiency (WUE) was calculated as the ratio A/E.

### 2.3. Measuring Malondialdehyde and H_2_O_2_ Concentration

Malondialdehyde (MDA) was measured to determine the lipid peroxidation in the hemp leaves. For MDA assay, the freeze-dried leaf samples (25 mg) were ground in 5 mL of 0.1% trichloroacetic acid and centrifuged at 10,000× *g* for 10 min at 4 °C. A 4 mL of 20% trichloroacetic acid (TCA) containing 0.5% thiobarbituric acid was added to 1 mL of supernatant. The mixture was heated at 95 °C for 30 min, followed by cooled quickly on an ice bath. The resulting mixture was centrifuged again at 5000 rpm for 15 min, and the absorbance was taken at 532 nm and 600 nm. An extinction coefficient of 155 mM^−1^ cm^−1^ was used to calculate the MDA concentration [47].

The H_2_O_2_ content was estimated according to the method developed by Singh et al. [48]. 25 mg of freeze-dried leaves were extracted in 5 mL of 0.1% (*w*/*v*) TCA and centrifuged at 12,000× *g* for 15 min in a refrigerated centrifuge. Then 0.5 mL of the supernatant was added to 0.5 mL of 10 mM potassium phosphate buffer (pH 7.0). After that, 1 mL of 1 M KI was added to the mixture and placed in a dark place (1 h) for incubation. The absorbance was measured at 390 nm, where a standard H_2_O_2_ curve was prepared to calculate the concentration of H_2_O_2_ in the sample.

### 2.4. Activities of Antioxidant Enzymes 

For the analysis of antioxidant enzymes, leaf samples were collected and immersed immediately in liquid nitrogen and stored at −80 °C until use. A 200 mg sample was homogenized in 5 mL of 50 mM sodium phosphate buffer solution (pH 7.8) using a pre-chilled mortar and pestle, then centrifuged at 15,000× *g* for 20 min at 4 °C. After collecting the supernatant, the enzyme extract was stored at 4 °C for analysis [49].

The superoxide dismutase activity (SOD; EC 1.15.1.1) was estimated by the method described earlier [50]. The reaction mixture for estimating SOD contained 50 mM sodium phosphate buffer with 0.1 mM EDTA, 12 mM methionine, 75 µM NBT, and 50 mM Na_2_CO_3_. Then, a 100 µL enzyme extract or 100 µL buffer was used in the sample or blank, respectively. After that, 300 µL of 0.1 mM Riboflavin was added to the reaction mixture to make 2 mL of the final volume. The tubes were shaken and irradiated under the fluorescent light (15 W) for 15 min. The absorbance was taken at 560 nm by a spectrophotometer. From the result, 50% inhibition of NBT reduction was considered as one unit of the enzyme [51].

The activities of Guaiacol peroxidase (POD; EC 1.11.1.7) and catalase (CAT; EC 1.11.1.6) were assayed by the method of Zhang [52]. For POD assay, a 3 mL reaction mixture contained 100 µL enzyme extract, 100 µL guaiacol (1.5%, *v/v*), 100 µL H_2_O_2_ (300 mM), and 2.7 mL 25 mM sodium phosphate buffer with 2 mM EDTA (pH 7.0). The absorbance was measured by a spectrophotometer at 470 nm (ɛ = 26.6 mM cm^−1^). On the other hand, The assay mixture for CAT contained 100 µL of enzyme extract, 100 µL of H_2_O_2_ (300 mM), and 2.8 mL of 50 mM phosphate buffer with 2 mM EDTA (pH 7.0). The decreased absorbance rate was measured at 240 nm (ɛ = 39.4 mM cm^−1^).

The Ascorbate peroxidase (APX; EC 1.11.1.11) activity was assayed by the method of Nakano and Asada [53]. The reaction mixture (3 mL) contained 25 µL enzyme extract, 100 µL ascorbate (7.5 mM), 100 µL H_2_O_2_ (300 mM), and 2.775 mL of 25 mM sodium phosphate buffer (2 mM EDTA, pH 7.0). The decrease of absorbance at 290 nm was considered to calculate APX activity using an extinction coefficient of 2.8 mM cm^−1^.

### 2.5. Estimation of Total Phenolic Content (TPC), Total Flavonoid Content (TFC), and Antioxidant Capacity

25 mg of freeze-dried leaf samples were homogenized in 10 mL of ethanol (80%, *v*/*v* in water) followed by sonication at 35 °C for 60 min. Then, the extracts were filtered (Advantech 5B filter paper, Tokoyo Roshi Kaisha Ltd., Saitama, Japan) and preserved at 4 °C in a refrigerator for further analysis).

The total phenolic content (TPC) of the sample was estimated by the Folin-Ciocalteu method [54]. The reaction mixture consisted of a 1 mL sample, 200 µL phenol reagent (1N), and 1.8 mL water. The mixture was vortexed and wait for 3 min, and then 400 µL of Na_2_CO_3_ (10%, *v/v* in water) was added. After that, the mixture was diluted by adding 600 µL of distilled water to get the final volume (4 mL) and left for 1 h incubation at room temperature. The absorbance was taken at 725 nm spectrophotometrically, and TPC was calculated based on a standard calibration curve of Gallic acid and expressed as µg g^−1^ dry weight.

The total flavonoid content (TFC) was determined by the method of Ghimeray et al. [55]. In the assay, 500 µL of the extract was mixed with 100 µL of Al(NO_3_)_3_ (10%, *w*/*v*) and 100 µL of potassium acetate (1 M) solution. After that 3.3 mL of distilled water was added to the mixture to adjust the final volume up to 4 mL. The reaction mixture was vortexed and incubated at room temperature for 40 min. Then the absorbance was measured at 415 nm by a UV-Vis spectrophotometer, and the TFC was calculated as mg/g of Quercetin equivalent on a dry weight basis.

The free radical scavenging activity of cannabis leaf was estimated by the method of Braca et al. [56]. Briefly, DPPH (2,2-diphenyl-1 picrylhydrazyl) powder (5.914 mg) was dissolved in methanol (100 mL) to prepare a stock solution, and the absorbance range was maintained between 1.1 and 1.3 by a spectrophotometer. Then 1 mL extract was mixed with 3 mL of DPPH in test tubes followed by vortexed and allowed to stand for 30 min at room temperature in the dark. The distilled water was used instead of the plant extract to prepare the blank sample. The absorbance was taken at 517 nm where the scavenging capacity of the samples was calculated by the formula given below, and results were expressed as a percentage (%):

Inhibition (%) = [(blank sample − extract sample)/blank sample] × 100

### 2.6. Determination of Tetrahydrocannabinol (THC), Tetrahydrocannabinolic Acid (THCA), Cannabidiol (CBD), and Cannabidiolic Acid (CBDA)

The freeze-dried (100 mg) leaf sample was dissolved in 5 mL of methanol (100%) and sonicated at room temperature for 20 min. After filtration through a syringe filter (0.45 µM, Millipore, Bedford, MA, USA), the solution was kept in a refrigerator at 4 °C. The HPLC system (Shimadzu LC-20 AT, Shimadzu Co., Ltd., Kyoto, Japan) with a UV-VIS detector and a reverse phase Zorbax SB-C18 column (4.6 mm × 100 mm, 3.5 µm, Agilent Technologies, Inc., Santa Clara, CA, USA) was used. The mobile phase was 70% acetonitrile containing 0.1% phosphoric acid with isocratic elution mode. The retention times of standard CBDA, CBD, Δ9-THC, and Δ9-THCA were 3.60, 4.34, 9.60, and 13.00 min, respectively (Supplementary Figure S1). A 10 µL sample was injected where the flow rate and oven temperature was 1.5 mL min^−1^ and 27 °C, respectively. The detection wavelength was used 275 nm with three biological replications.

### 2.7. Statistical Analysis

All results were expressed as mean ± SE (standard error). The one-way analysis of variance was performed using Statistix 10 (Tallahassee, FL 32312, USA) following Complete Randomized Design (CRD). Different letters indicate the statistically significant differences between treatments at *p* < 0.05, according to the least significant differences (LSD). The heatmap and clustering analysis were prepared by MetaboAnalyst 4.0 (www.metaboanalyst.ca, accessed on 19 July 2021) [57], where samples were normalized by sum, and auto-scaling features were applied. The hierarchical cluster analysis was conducted using the Euclidean distance metric (average linking clustering). The principal component analysis (PCA) was carried out using OriginLab 10.0 software (OriginLab, Northampton, MA, USA). The correlation test was done by SPSS statistical software package (Ver. 23.0, SPSS Inc., Chicago, IL, USA).

## 3. Results and Discussion

### 3.1. Effect of LED on Photosynthetic Gas Exchange

**Figure 2:**
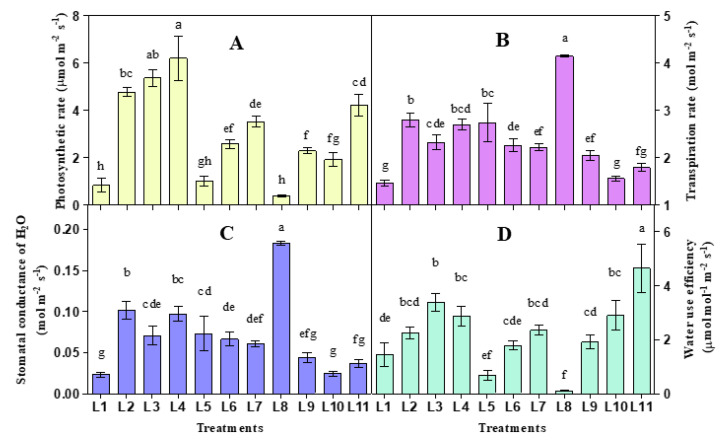
Effect of 20 days exposure to different LED spectra on photosynthetic rate (**A**), Transpiration rate (**B**), Stomatal conductance (**C**) and Water use efficiency (**D**) of hemp seedlings. Here and subsequent figures: L1, Natural light; L2, White; L3, R_8_:B_2_; L4, R_7_:B_2_:G_1_; L5, R_7_:B_2_:FR_1_; L6, R_6_:B_2_:G_1_:FR_1_; L7, R_5_:B_2_:W_2_:FR_1_; L8, R_5_:B_2_:G_1_:FR_1_:UV_1_; L9, R_6_:B_2_:FR_1_:UV_1_; L10, R_4_:B_2_:W_2_:FR_1_:UV_1_; L11, R_2_:B_2_:G_2_:W_2_:FR_1_:UV_1_. All treatments used a photosynthetic photon flux density of 300 µmol m^−2^ s^−1^. Vertical bars indicate mean ± SE of six replicates. Different letters indicate significant differences at *p* < 0.05.

Photosynthetic parameters varied concerning different light treatments (Figure 2). In the current study, photosynthetic rate, transpiration rate, stomatal conductance and water use efficiency ranged from 0.397–6.23 µmol m^−2^ s^−1^, 0.587–3.942 mol m^−2^ s^−1^, 0.023–0.1833 mol m^−2^ s^−1^ and 0.101–4.647 µmol mol m^−2^ s^−1^, respectively. The higher photosynthetic rate was observed in L4, while the transpiration rate and stomatal conductance were higher in L8 and L2 light treatments. On the other hand, higher water use efficiency was recorded in L11 treatment.

Photosynthesis can be affected by the stomatal density, distribution, and opening status as it regulates the diffusion of water vapor and the uptake of carbon dioxide in plants. Besides, many factors can influence stomatal behavior, including light, CO_2_ concentration, and temperature [58]. Some previous studies suggested that light intensity can enhance stomatal conductance in plants [59,60,61]. Simultaneously, both lower and excessive light can reduce the photosynthetic rate and stomatal conductance [61]. It has also been known that the photosynthetic rate depends on chlorophyll content, and it can be affected by any change in it [62,63]. Our study also presented a similar result as under the treatments L1, L5, L6, L7, L8, and L9 plants attain lower chlorophyll content and lower photosynthetic rate. We also observe a similar pattern of results between photosynthesis and water use efficiency and stomatal conductance and transpiration rate.

Both photosynthetic rate and water use efficiency were increased under all light treatments except L5 and L8. On the other hand, transpiration and stomatal conductance significantly increased under all light spectra compared to natural light, except L9, L10 and L11. Plant attained a higher photosynthetic rate and water use efficiency and lower transpiration rate and stomatal conductance under L3, L4, and L11. On the other hand, photosynthetic rate and water use efficiency, and higher transpiration rate and stomatal conductance were recorded lower in L5 and L8 treatments.

### 3.2. Influence of LED on Lipid Peroxidation and Hydrogen Peroxide

**Figure 3:**
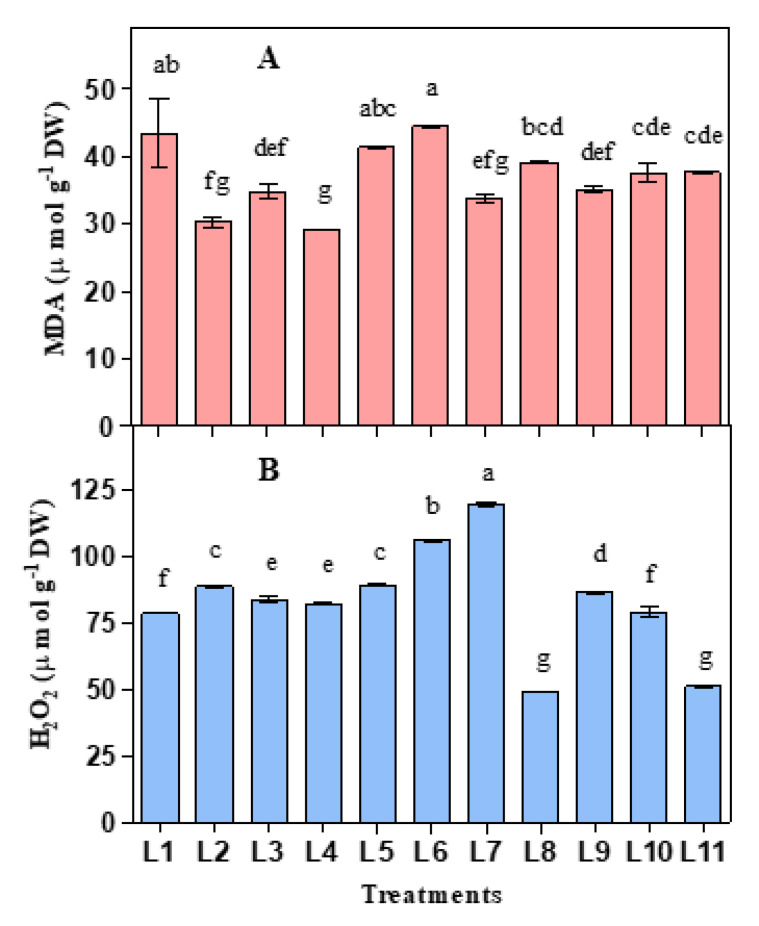
Effect of 20 days exposure to different LED spectra on Lipid peroxidation (MDA) (**A**) and H_2_O_2_ (**B**) of hemp seedlings. Here and subsequent figures: L1, Natural light; L2, White; L3, R_8_:B_2_; L4, R_7_:B_2_:G_1_; L5, R_7_:B_2_:FR_1_; L6, R_6_:B_2_:G_1_:FR_1_; L7, R_5_:B_2_:W_2_:FR_1_; L8, R_5_:B_2_:G_1_:FR_1_:UV_1_; L9, R_6_:B_2_:FR_1_:UV_1_; L10, R_4_:B_2_:W_2_:FR_1_:UV_1_; L11, R_2_:B_2_:G_2_:W_2_:FR_1_:UV_1_. All treatments used a photosynthetic photon flux density of 300 µmol m^−2^ s^−1^. Vertical bars indicate mean ± SE of four replicates. Different letters indicate significant differences at *p* < 0.05.

Both malondialdehyde (MDA) and H_2_O_2_ level were considerably influenced by different light treatments (Figure 3). Higher MDA was recorded in L6, followed by L1, L5, and L8, while lower MDA was observed in L2, L3, and L4 treatments. On the other hand, plants accumulated higher H_2_O_2_ in L7, followed by L6, L5, L2, and L9, while lower H_2_O_2_ was observed in L8 and L11 treatments.

In the presence of light, chloroplasts and peroxisomes act as leading ROS producers in plants [64]. Thylakoids are the membrane-bound compartments inside chloroplasts that harbors the efficient light for light-dependent photosynthesis reactions by PS I and PS II [65,66]. Light energy at the over-saturation point is responsible for photoinhibition by reducing the light-induced photochemical activity in PS II. These negative changes in the photosynthetic electron transport system are mainly responsible for the generation of ROS [12,13,67]. In these connections during overexcitation of chlorophyll, ^1^O_2_ and O_2_^−^^•^ produce from O_2_ in PS II (during electron transfer to O_2_ through Q_A_ and Q_B_) and PS I (Mehler reaction), respectively [68,69,70]. Peroxisomes can generate H_2_O_2_ by the activities of flavin oxidase, while O_2_^−^^•^ and H_2_O_2_ may be generated in mitochondria of the cell by reducing O_2_ near the electric transport chain [71,72,73]. In the present experiment, under the light treatments, L5, L6, L7, and L9 accumulated higher H_2_O_2_ with a lower photosynthetic rate indicating an active production of ROS resulting in photoinhibition and/or overexcitation of chlorophyll. To scavenge the excess ROS produced in the electron transport system plant uses various antioxidative defense mechanisms, including enzymatic and non-enzymatic scavenging procedures, which work synergistically and interactively with each other [74,75].

Lipids and proteins are the primary victims of oxidative damage by ROS accumulated in plant cells [76]. Lipid peroxidation, considered as an indicator of determining the lipid damage extent, occurs in every organism by the oxidative decomposition of polyunsaturated lipids in the plasma membrane under severe conditions [77,78,79]. However, constant stress for plant generates redundant ROS that cannot be entirely homeostated by the scavenging system of the cell and exert some physiological actions like lipid peroxidation, nucleic acid oxidation, protein denaturation, enzyme activity inhibition and finally lead to programmed cell death [69,76,80]. In the present study, under the light treatments, L1, L5, L6, and L8 produced higher MDA along with lower photosynthetic rate and water use efficiency, indicating severe lipid damage in the plasma membrane of the plant cell.

### 3.3. Effect of LED Spectra on Antioxidant Enzymes Activities

**Figure 4:**
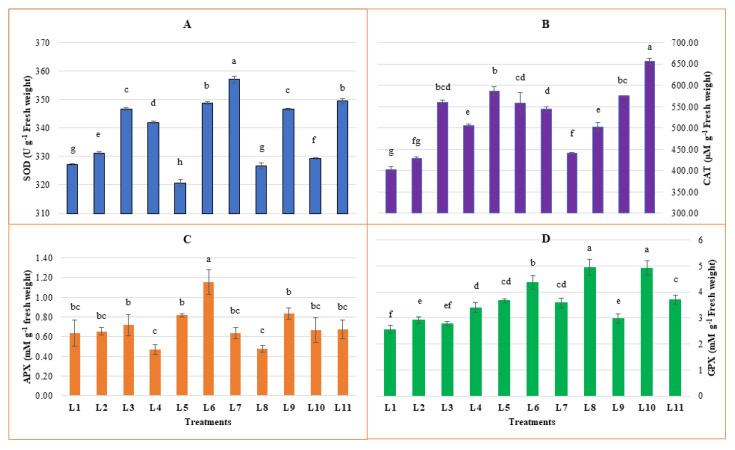
Effect of different LED spectra on Superoxide dismutase (SOD) (**A**), Catalase (CAT) (**B**), Ascorbate peroxidase (APX) (**C**) and Guaiacol peroxidase (GPX) (**D**) for 20 days on hemp seedlings. Here and subsequent figures: L1, Natural light; L2, White; L3, R_8_:B_2_; L4, R_7_:B_2_:G_1_; L5, R_7_:B_2_:FR_1_; L6, R_6_:B_2_:G_1_:FR_1_; L7, R_5_:B_2_:W_2_:FR_1_; L8, R_5_:B_2_:G_1_:FR_1_:UV_1_; L9, R_6_:B_2_:FR_1_:UV_1_; L10, R_4_:B_2_:W_2_:FR_1_:UV_1_; L11, R_2_:B_2_:G_2_:W_2_:FR_1_:UV_1_. All treatments used a photosynthetic photon flux density of 300 µmol m^−2^ s^−1^. Vertical bars indicate mean ± SE of four replicates. Different letters indicate significant differences at *p* < 0.05.

From our study, the highest and lowest SOD activity was recorded in L7 and L5 treatments, respectively (Figure 4). However, a higher increment was observed from L7 (9.1%), followed by L11 (6.76%), L6 (6.58%), L3 (5.94%), and L9 (5.89%), respectively compared to natural light. Higher CAT was recorded in L11 followed by L5, L10, L3, L6, and L7 with 62.88%, 45.49%, 42.66%, 39.11%, 38.49% and 34.9% increment (compared to natural light), respectively. Higher APX was recorded in L6, followed by L9, L5, L3, and L11, with 81.12%, 30.77%, 27.97%, 12.59%, and 5.59% increment, respectively, with comparison to natural light. On the other hand, a higher reduction of APX activity was also observed in L4 (26.57%) and L8 (25.17%). However, higher activity of GPX was observed in L8, L10, L6, L11, L5, and L7 with 92.6%, 91.6%, 70.32%, 44.3%, 42.8%, and 39.4% increment, respectively compared to natural light.

ROS accumulated under stress conditions can act as signaling molecules and trigger a signal transduction pathway. It is also crucial that despite causing programmed cell death, ROS is inevitable to confer the resistance to stress. Notably, the activated response created by ROS should be rapid and decay as long as the stress disappeared [75]. The main antioxidant enzymes that play a vital role in detoxifying ROS are SOD, CAT, and APX. SOD converts O_2_^−^ to O_2_ and H_2_O_2_, while CAT, APX, and other peroxidase convert H_2_O_2_ to H_2_O and O_2_ [19,81]. In the present experiment in L6 and L7 light treatment, both H_2_O_2_ accumulation and SOD activity was higher, indicating an active mode of stress and plant response to mitigate the ROS compound. Under the light treatment, L1, L5, L6, L8, L10, and L11 plant accumulated higher MDA indicated a secondary damage occurrence is running by lipid peroxidation in the plant cell. At the same time, higher activity of CAT in L3, L5, L6, L10, and L11, higher APX activity from L3, L5, L6 and L9, higher GPX activity from L5, L6, L8 and L10 light spectra were recorded. On the other hand, activity of SOD was found lower in L1, L5, L8 and L10 treatments. Earlier, a decreased SOD and increased APX activity with the increasing MDA accumulation under drought stress were reported [82]. These results indicate that lipid peroxidation may be activated with the lower activity of SOD and higher activity of peroxidases. Generally, elevated oxidative stress stimulates the production of H_2_O_2_ and provokes the increase of antioxidant enzyme activities, which help minimize the negative effect of abiotic stress [83]. A previous study found that a higher irradiance of far-red and red light treatment plants produces higher MDA than lower irradiance [20]. Higher MDA from L5, L6, L8, L10, and L11 compared to other LED spectra in the present study may be due to the presence of far-red light in those spectra.

### 3.4. Effect of LED Spectra on Antioxidant Activities

**Figure 5:**
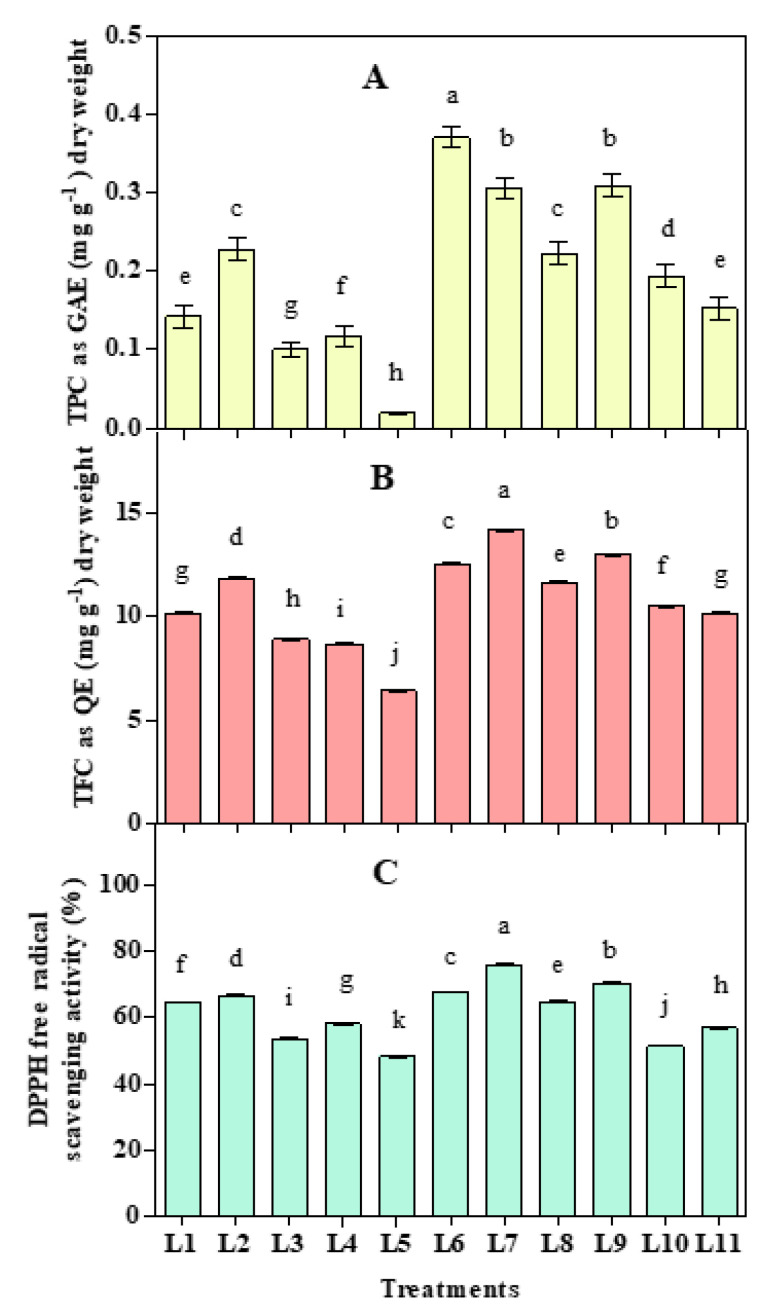
Effect of 20 days exposure to different LED spectra on Total polyphenol (TPC) (**A**), Total flavonoid (TFC) (**B**), and DPPH radical scavenging activity (**C**) of hemp seedlings. Here and subsequent figures: L1, Natural light; L2, White; L3, R_8_:B_2_; L4, R_7_:B_2_:G_1_; L5, R_7_:B_2_:FR_1_; L6, R_6_:B_2_:G_1_:FR_1_; L7, R_5_:B_2_:W_2_:FR_1_; L8, R_5_:B_2_:G_1_:FR_1_:UV_1_; L9, R_6_:B_2_:FR_1_:UV_1_; L10, R_4_:B_2_:W_2_:FR_1_:UV_1_; L11, R_2_:B_2_:G_2_:W_2_:FR_1_:UV_1_. All treatments used a photosynthetic photon flux density of 300 µmol m^−2^ s^−1^. Vertical bars indicate mean ± SE of four replicates. Different letters indicate significant differences at *p* < 0.05.

Total polyphenol (TPC) and total flavonoid (TFC) varied with the spectral variation (Figure 5). Higher TPC was recorded in L6, while both TFC and DPPH free radical scavenging activity (%) was recorded higher in L7 treatment. Results also showed that both TPC and TFC increased under L2, L6, L7, L8, L9, and L10 treatments compare to natural light, while DPPH free radical scavenging activity (%) increased under L2, L6, L7, L8, and L9 treatments.

Generally, the cytokinin level increased by a red light that can stimulate the synthesis of phenolics compound, where far-red helps increase the plants’ antioxidant capacity [84,85]. A previous study of both phenolic compound and antioxidant capacity decreased under a combination of red and blue compared to monochromatic red, blue, and natural light [18]. Therefore, the intensity of red light and its ratio with other light sources may contribute to secondary metabolites production. Further, secondary metabolites and antioxidant capacities can vary with the light intensities and ratio of monochromatic light sources [86,87,88]. In our study, both TPC and TFC decreased at ≥70% and increased at 50–60%, while it turned to dropped at <40% red light sources compare to natural light. Supplementary UV radiation can increase flavanols and other secondary metabolites that act as a stress response to protect plants from radiation [17,89]. In our study, UV A radiation was observed prominent with a 60% red light source. Artificial blue LED and far-red light enhance secondary metabolites, and the nutritional quality of crops, including ascorbate, total phenolic compounds, total flavonoid contents, and antioxidant activity, have been reported [88,90]. We also found an increment of secondary metabolites with the addition of FR light, but the effect of FR light was found prominent with 50–60% red light sources. A previous study stated that increasing intensity of red to blue increased plant flavonoid, which was found best at 7:3 ratio [87]. Our research also produces higher flavonoids at L6, L7, L8, and L9 treatments with similar red and blue ratios.

### 3.5. Effect of LED Spectra on THC, THCA, CBD, and CBDA

**Figure 6:**
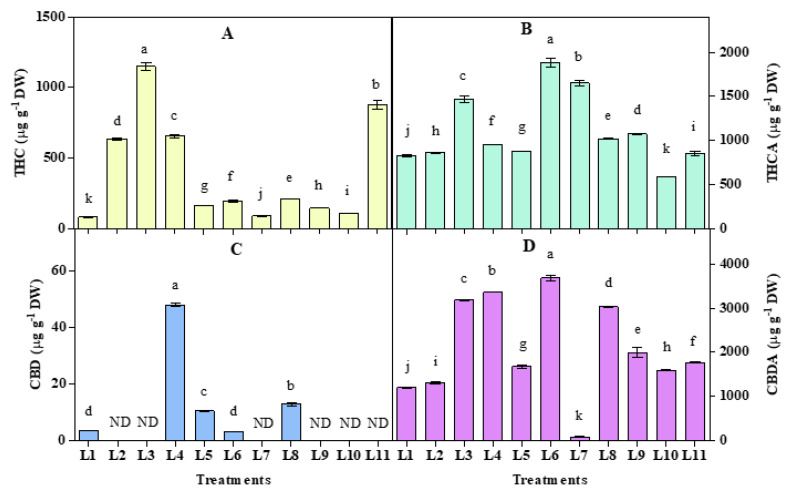
Effect of 20 days exposure to different LED spectra on Tetrahydrocannabinol (THC) (**A**), Tetrahydrocannabinolic acid (THCA) (**B**) Cannabidiol (CBD) (**C**), and Cannabidiolic acid (CBDA) (**D**) of hemp seedlings. Here and subsequent figures: L1, Natural light; L2, White; L3, R_8_:B_2_; L4, R_7_:B_2_:G_1_; L5, R_7_:B_2_:FR_1_; L6, R_6_:B_2_:G_1_:FR_1_; L7, R_5_:B_2_:W_2_:FR_1_; L8, R_5_:B_2_:G_1_:FR_1_:UV_1_; L9, R_6_:B_2_:FR_1_:UV_1_; L10, R_4_:B_2_:W_2_:FR_1_:UV_1_; L11, R_2_:B_2_:G_2_:W_2_:FR_1_:UV_1_. All treatments used a photosynthetic photon flux density of 300 µmol m^−2^ s^−1^. Vertical bars indicate mean ± SE of four replicates. Different letters indicate significant differences at *p* < 0.05.

Significant (*p* < 0.05) variations in the Tetrahydrocannabinol (THC), Tetrahydrocannabinolic acid (THCA), Cannabidiol (CBD), and Cannabidiolic acid (CBDA) were observed under different LED spectra (Figure 6). Plant accumulated higher CBD in L4 (48.01 µg g^−1^ DW), L5 (10.44 µg g^−1^ DW), and L8 (12.90 µg g^−1^ DW) while higher THC in all light spectra compare to natural light. Notably, CBD and THC showing a positive relationship in L4, L5, and L8 spectra, where both CBD and THC increased significantly. On the other hand, an opposite relationship was observed in L2, L3, L7, L9, L10, and L11 spectra, where THC and CBD showed an increasing and decreasing trend, respectively. Higher CBDA was accumulated under all spectra except L7, and higher THCA was accumulated under all spectra except L10 compared to natural light. Interestingly, L7 produced an antagonistic relationship, while others produced an almost positive relationship between THCA and CBDA accumulation.

**Figure 7:**
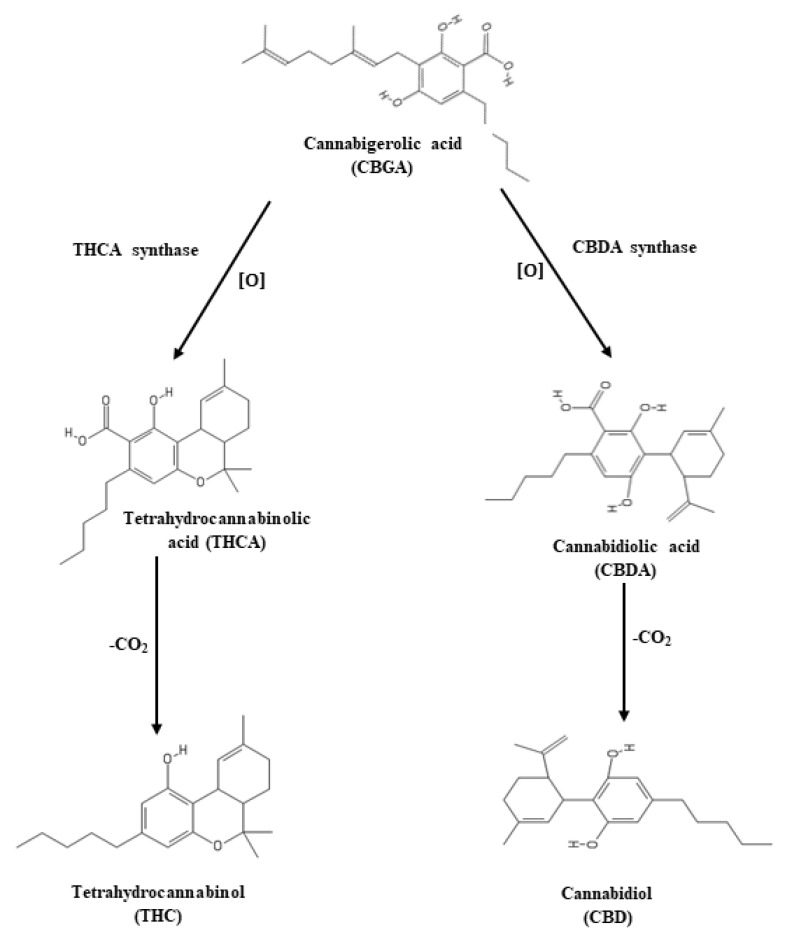
Biosynthetic pathway of THC and CBD formation. THCA synthase and CBDA synthase catalyze oxidative cyclization of the monoterpene moiety of CBGA to form THCA and CBDA. THC and CBD are derived by non-enzymatic decarboxylation of THCA and CBDA.

In general, CBGA produces by alkylation of two precursors olivetolic acid and geranyl pyrophosphate, with the help of geranyl pyrophosphate:olivatolate geranyl transferase [91,92], which further can convert to THCA by THCA synthase [93,94] and CBDA by CBDA synthase [95] in the oxidation process (Figure 7). In this connection, during oxidation of CBGA, it produces hydrogen peroxide and THCA in THCA synthase reaction [96], which may play a role in the self-defense of cannabis plants [94]. Furthermore, light quality may play an essential role in cannabinoid synthesis as light intensity influences cannabis yields strongly [41,97]. We observed both higher THCA and H_2_O_2_ accumulation in L6 and L7 spectra in the present study, but we did not find any clear relation between THC and H_2_O_2_ from this observation. THCA also showed a positive relationship with antioxidant activities and antioxidant enzymes in L6, L7, L8, and L9 treatments.

On the other hand, it showed a negative relationship with the photosynthetic rate in the above four treatments. In the present study, except L11, THC accumulation was most prominent in L2 (white), L3 (R_8_B_2_), and L4 (R_7_B_2_G_1_) spectra, where we can assume very little influence of FR and UV A light on THC accumulation in cannabis plants. It was also reported that Cannabis plants were grown under blue, and synergy between UV-A and blue light improved cannabinoid and cannabigerol accumulation, respectively [98]. We also found higher THCA, CBDA, and THC concentrations under UV-A mediated spectral combinations.

On the other hand, CBD and CBDA accumulated higher in L4, L5, L6, and L8. From these results, we can see that green light has a significant role in CBDA synthesis and its conversion to CBD. Notably, FR light also influences CBDA and CBD accumulation along with green light, where white and UV-A play a negative role in this process. In some previous studies, the role of green light was shown negative for THC accumulation [7,99], but its role in CBD and CBDA synthesis was not clear. Results also depicted that the total THC (THC + THCA) was accumulated higher in L3, L6, L7, and L11, while total CBD (CBD + CBDA) was recorded higher in L3, L4, L6, and L8 treatments (Supplementary Figure S2). However, due to their importance, higher THC accumulation in L2, L3, L4, and L11 and higher CBD in L4 and L8 treatments got more attention in this study. Despite having some shreds of evidence in the previous studies [46,100,101], the complex functions of cannabinoids relate to the defensive role toward biotic and abiotic stresses are not clear. Among the cannabinoids, THC and CBD were most discussed for having their antioxidant properties [102]. Earlier, THC, THCA, CBD, and CBDA were predicted as stress indicators along with some other secondary metabolites in the hemp plant under controlled drought stress [100]. It was also reported that THCA induces necrotic cell death in Cannabis cells and leaves [103]. The increasing cannabinoids in the present study also indicated a stress response of the cannabis plant under some controlled LED light spectral treatments.

### 3.6. Hierarchical Clustering and Heatmap Unveiled the Connections between Variables and Treatments

**Figure 8:**
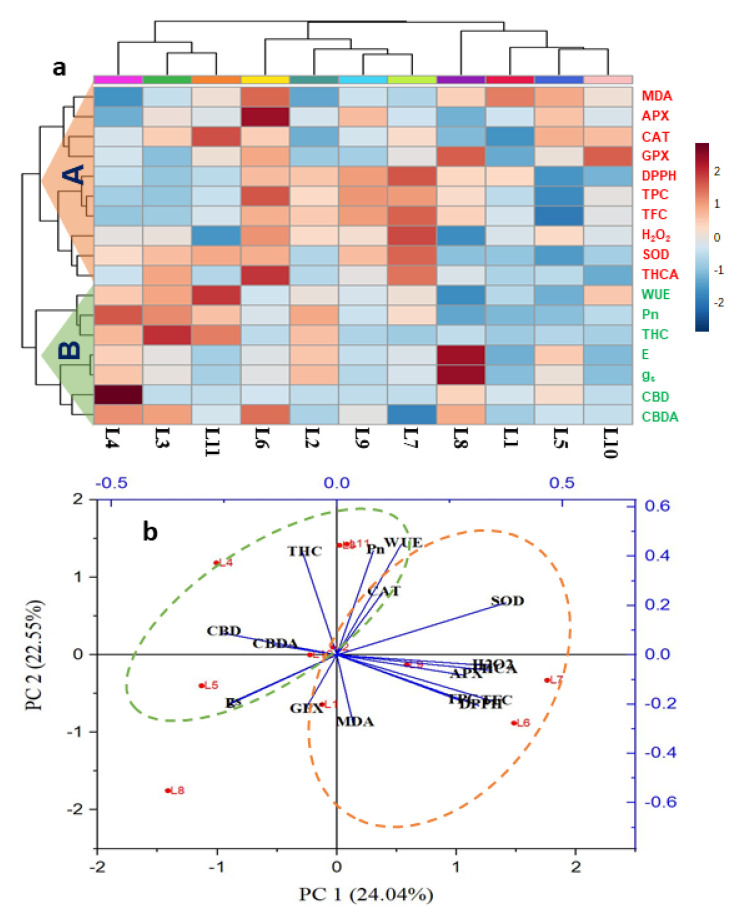
Hierarchical clustering and heatmap analysis (**a**) and principal component analysis (PCA) (**b**) to elucidate the variable treatment relationships under eleven treatments for 20 days. In the heatmap, the mean values of the various parameters obtained in this study were normalized and clustered. At the variable level, two major clusters were recognized for each treatment. The color scale displays the intensity of normalized mean values of different parameters. In PCA, the lines starting from the central point of the biplots display negative or positive associations of different variables, and their proximity specifies the degree of correlation with specific treatment. L1, Natural light; L2, White; L3, R_8_:B_2_; L4, R_7_:B_2_:G_1_; L5, R_7_:B_2_:FR_1_; L6, R_6_:B_2_:G_1_:FR_1_; L7, R_5_:B_2_:W_2_:FR_1_; L8, R_5_:B_2_:G_1_:FR_1_:UV_1_; L9, R_6_:B_2_:FR_1_:UV_1_; L10, R_4_:B_2_:W_2_:FR_1_:UV_1_; L11, R_2_:B_2_:G_2_:W_2_:FR_1_:UV_1_. Pn, photosynthetic rate; E, transpiration rate; gs, stomatal conductivity; WUE, water use efficiency; MDA, malondialdehyde; H_2_O_2_, hydrogen peroxide; SOD, superoxide dismutase; CAT, catalase; APX, ascorbate peroxidase; GPX, guaiacol peroxidase; TPC, total polyphenol; TFC, total flavonoid; DPPH, DPPH radical scavenging activity; CBD, cannabidiol; CBDA, cannabidiolic acid; THC, Tetrahydrocannabinol; THCA, Tetrahydrocannabinolic acid.

The values of all physiological and biochemical parameters of different light treatments were employed to construct the hierarchical clustering and a heatmap (Figure 8a). Hierarchical clustering grouped all the variables into two major clusters (cluster-A and cluster-B). Hierarchical clustering and heatmap revealed that all the parameters characterized cluster-A relate to abiotic stress, such as MDA, H_2_O_2_, SOD, CAT, APX, GPX, TPC, TFC, DPPH, and THCA. All the cluster-A variables showed minimal values at L1, L2, L3, and L4, which indicated low comparative stress for hemp, whereas L6 and L7 treatments increased this trend. On the other hand, cluster-B represents all photosynthetic attributes (P_n_, E, g_s,_ and WUE) and cannabinoids like CBD, CBDA, and THC. All cluster-B variables showed maximum values at L4 followed by L8, L2, and L3. This result is indicating that CBD and THC have a negligible relationship with stress-producing compounds. On the other hand, CBDA has a small extent of the relationship with stress compounds as it increased a little at stress-producing light like L6. Interestingly, the treatment L4 exhibited minimum and maximum values of almost all cluster-A and cluster-B parameters, respectively.

PCA analysis was carried out to uncover the connection of the different parameters with different treatment groups (Figure 8b). The two elements of PCA (PC1 and PC2) together described 46.59% of data variability. The results demonstrated an intimate association of cluster A variables with mainly L6 and L7 treatments. Results also displayed that some variables related to stress responses (H_2_O_2_, MDA, TPC, TFC, DPPH, SOD, and APX) were negatively correlated to CBD, and CBDA while THC maintained a positive correlation in most cases, which is also confirmed by bivariate correlation (Supplementary Table S1). Besides, treatment L4 showed an intimate relationship with THC, CBD, and CBDA.

## 4. Conclusions

Higher CBD was accumulated in L4 (R_7_:B_2_:G_1_), L5 (R_7_:B_2_:FR_1_), and L8 (R_5_:B_2_:G_1_:FR_1_:UV_1_), while higher THC in all light spectra compare to natural light. On the other hand, higher CBDA synthesis was recorded in L3 (R_8_:B_2_), L4 (R_7_:B_2_:G_1_), L6 (R_6_:B_2_:G_1_:FR_1_), and L8 (R_5_:B_2_:G_1_:FR_1_:UV_1_) treatments. The treatment L4 (R_7_:B_2_:G_1_) produced all cannabinoids (CBD, CBDA, THC, and THCA) in higher concentration with lower stress response compounds like reactive oxygen species, antioxidants, THCA, and enzymatic activities. Besides this, the treatments L6 (R_6_:B_2_:G_1_:FR_1_) showed a lower CBD and THC and higher THCA and CBDA accumulation with higher activities of all other stress response compounds. On the other hand, L7 (R_5_:B_2_:W_2_:FR_1_) produced lower CBD, THC, and CBDA with a higher accumulation of all other stress-responsive compounds, including THCA. Besides, Greenlight has a significant role in CBD and CBDA synthesis where FR and UV-A (along with green) play a positive and negative role in this process, respectively. From our result, THCA showed a significant role as a stress marker followed by CBDA. On the other hand, THC and CBD showed a negligible response as stress response compounds to such conditions.

## Figures and Tables

**Table 1:** LED light composition.

Spectrum Combinations	Ratio (%)	Intensity (µmol m^−2^ s^−1^)	Code Name
Natural light	-	-	L1
White	100	300	L2
R + B	80:20	300	L3
R + B + G	70:20:10	300	L4
R + B + FR	70:20:10	300	L5
R + B + G + FR	60:20:10:10	300	L6
R + B + W + FR	50:20:20:10	300	L7
R + B + G + FR + UV	50:20:10:10:10	300	L8
R + B + FR + UV	60:20:10:10	300	L9
R + B + W + FR + UV	40:20:20:10:10	300	L10
R + B + G + W + FR + UV	20:20:20:20:10:10	300	L11

**Table 2:** Nutrient solution.

Chemical Name	A Tank (50 L) *	B Tank (50 L)
Ca(NO_3_)	1.5 kg	
KNO_3_	3.79 kg	3.79 kg
(NH_4_)_2_HPO_4_		1.6 kg
MgSO_4_		4.3 kg
K_2_SO_4_		
Fe-EDTA	460 g	
MnSO_4_		30.8 g
H_3_BO_3_		57.2 g
ZnSO_4_		3.6 g
CuSO_4_		1.3 g
(NH_4_)_6_Mo_7_O_24_.4H_2_O		0.4 g

* Solution of Tank A and Tank B were subjected to mixed to maintain a E.C. range between 1.2–1.7 (ds m^−2^).

## Data Availability

Not applicable.

## Supplementary Materials

The following are available online at https://www.mdpi.com/article/10.3390/biology10080710/s1, Figure S1: The retention time of CBD (4.34 min), CBDA (3.60 min), THC (9.60 min), and THCA (13.00 min), Figure S2: Effect of 20 days exposure to different LED spectra on total THC (THC + THCA; A), and total CBD (CBD + CBDA; B) of hemp seedlings, Table S1: The bivariate correlations among the parameters under 11 treatments.
