# Supplementary material for: Cannabinoids Accumulation in Hemp (Cannabis sativa L.) Plants under LED Light Spectra and Their Discrete Role as a Stress Marker
Source: Biology (Basel). 2021 Jul 24;10(8):710. doi: 10.3390/biology10080710 (PMC8389281; doi:10.3390/biology10080710)
Supplement: Supplementary file 1 [file biology-10-00710-s001.zip › biology-1215694-supplementary.pdf]

biology

MDPI

Article

Supplementary Materials: Cannabinoids Accumulation in Hemp (*Cannabis Sativa* L) Plants Under LED Light Spectra and Their Discrete Role as A Stress Marker

Md. Jahirul Islam, Byeong Ryeol Ryu, Md. Obyedul Kalam Azad, Md. Hafizur Rahman, Eun Ju Cheong, Jung-Dae Lim, and Young-Seok Lim

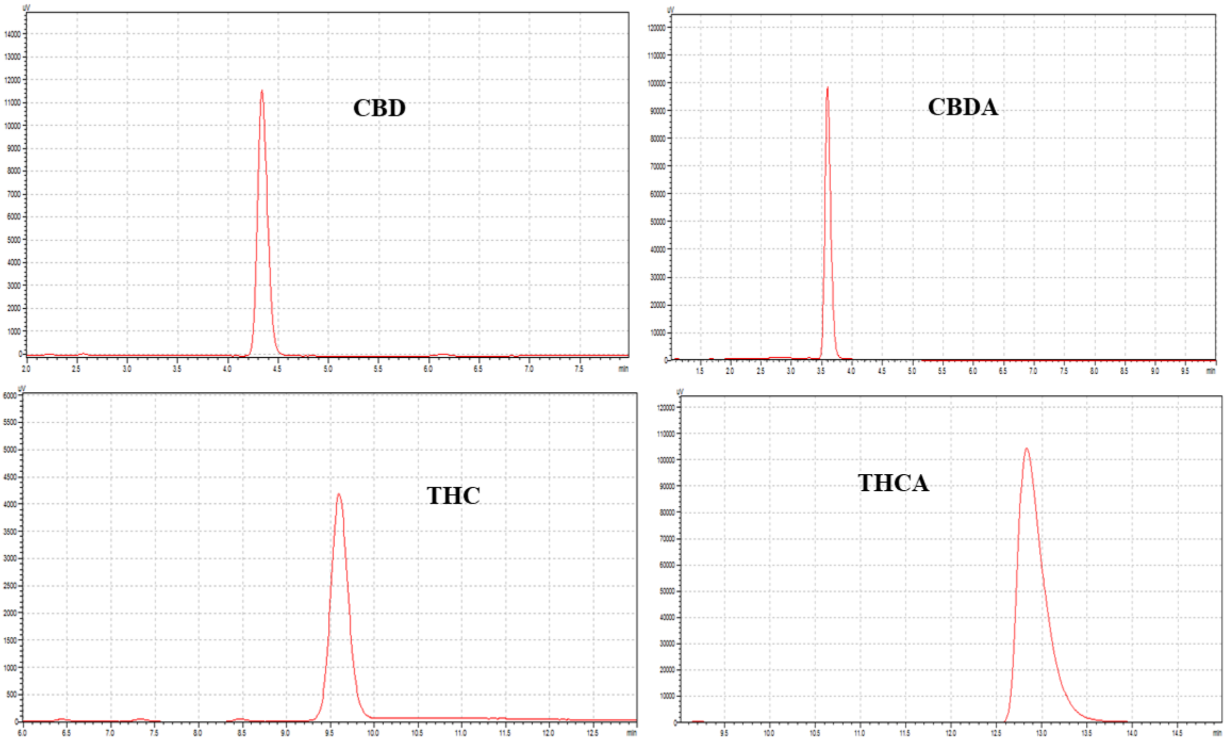

Figure S1. The retention time of CBD (4.34 min), CBDA (3.60 min), THC (9.60 min), and THCA (13.00 min).

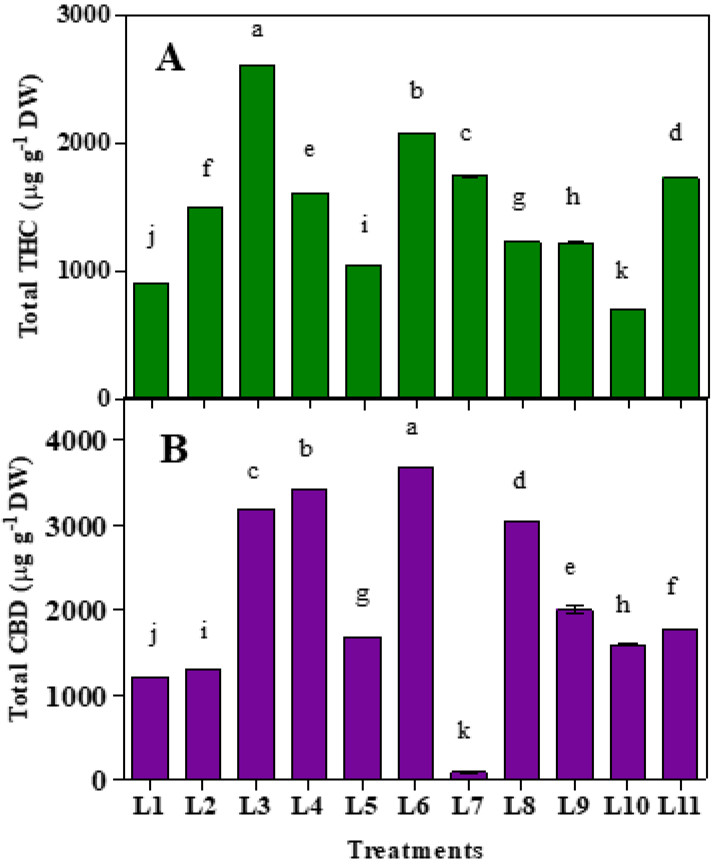

Figure S2. Effect of 20 days exposure to different LED spectra on total THC (THC + THCA; **A**), and total CBD (CBD + CBDA; **B**) of hemp seedlings. Here and subsequent figures: L1, Natural light; L2, White; L3, R_8:B_2; L4, R_7:B_2:G_1; L5, R_7:B_2:FR_1; L6, R_6:B_2:G_1:FR_1; L7, R_5:B_2:W_2:FR_1; L8, R_5:B_2:G_1:FR_1:UV_1; L9, R_6:B_2:FR_1:UV_1; L10, R_4:B_2:W_2:FR_1:UV_1; L11, R_2:B_2:G_2:W_2:FR_1:UV_1. All treatments used a photosynthetic photon flux density of 300 μmol m^{-2} s^{-1}. Column height indicates mean, vertical bars indicate standard error means (n = 3), and different letters indicate significant differences at $p < 0.05$ based on Duncan's multiple range test.

Table S1. The bivariate correlations among the parameters under 11 treatments.

	Pn	E	g_s	WUE	MDA	H_2O_2	SOD	CAT	APX	GPX	TPC	TFC	DPPH	THC	CBD	THCA	CBDA
**Pn**	1																
**E**	-0.078	1															
**g_s**	-0.023	0.985**	1														
**WUE**	0.699**	-0.552**	-0.507**	1													
**MDA**	-0.618**	-0.132	-0.165	-0.299*	1												
**H_2O_2**	0.163	-0.248	-0.280	-0.076	-0.072	1											
**SOD**	0.537**	-0.264	-0.233	0.526**	-0.236	0.378*	1										
**CAT**	0.221	-0.301*	-0.363*	0.515**	-0.063	0.013	0.393**	1									
**APX**	-0.116	-0.230	-0.265	-0.008	0.202	0.353*	0.180	0.299*	1								
**GPX**	-0.333*	0.287	0.282	-0.157	0.202	-0.209	-0.173	0.277	0.124	1							
**TPC**	-0.079	-0.031	0.011	-0.063	0.041	0.378*	0.491**	-0.131	0.369*	0.274	1						
**TFC**	-0.055	-0.029	0.024	-0.023	-0.079	0.324*	0.514**	-0.236	0.131	0.125	0.921**	1					
**DPPH**	-0.031	0.070	0.115	-0.180	-0.095	0.402**	0.478**	-0.443**	0.062	-0.149	0.789**	0.894**	1				
**THC**	0.756**	0.044	0.086	0.616**	-0.398**	-0.304*	0.298*	0.279	-0.144	-0.323*	-0.375*	-0.340*	-0.351*	1			
**CBD**	0.311*	0.352*	0.373*	-0.079	-0.305*	-0.121	-0.110	-0.140	-0.337*	0.042	-0.325*	-0.406**	-0.195	0.119	1		
**THCA**	0.179	0.084	0.087	-0.030	0.117	0.608**	0.674**	0.141	0.415**	-0.009	0.534**	0.444**	0.496**	0.027	-0.149	1	
**CBDA**	0.183	0.364*	0.395**	-0.041	0.085	-0.268	0.039	0.053	0.155	0.223	-0.018	-0.256	-0.260	0.359*	0.455*	0.260	1

*. Correlation is significant at the 0.05 level; **. Correlation is significant at the 0.01 level.
